# Evaluation of Nociception Using Quantitative Pupillometry and Skin Conductance in Critically Ill Unconscious Patients: A Pilot Study

**DOI:** 10.3390/brainsci11010109

**Published:** 2021-01-15

**Authors:** Sara Fratino, Lorenzo Peluso, Marta Talamonti, Marco Menozzi, Lucas Akira Costa Hirai, Francisco A Lobo, Chiara Prezioso, Jacques Creteur, Jean-François Payen, Fabio Silvio Taccone

**Affiliations:** 1 Department of Intensive Care, Erasme Hospital, Université Libre de Bruxelles, Route de Lennik 808, 1070 Brussels, Belgium; sarafratino@gmail.com (S.F.); marty.talamonti@gmail.com (M.T.); marc.menoz@gmail.com (M.M.); lucasakirahirai@gmail.com (L.A.C.H.); c.prezioso89@gmail.com (C.P.); jacques.creteur@erasme.ulb.ac.be (J.C.); ftaccone@ulb.ac.be (F.S.T.); 2 Department of Anesthesiology, CHTMAD-Hospital de S. Pedro, 5000 Vila Real, Portugal; xlanesth@gmail.com; 3 Department of Anesthesia and Critical Care CHU Grenoble Alpes, University Grenoble Alpes, 38000 Grenoble, France; Jean-Francois.Payen@ujf-grenoble.fr

**Keywords:** pain, automated pupillometer, critically illness, skin conductance, brain injury, algesimeter, analgesia

## Abstract

Background: Pain assessment is a challenge in critically ill patients, in particular those who are unable to express movements in reaction to noxious stimuli. The purpose of the study was to compare the pupillary response and skin conductance to pain stimulation in critically ill unconscious patients. Methods: This observational study included adult patients admitted to the intensive care unit (ICU) with acute brain injury (Glasgow Coma Scale < 9 with a motor response < 5) and/or requirements for deep level of sedation. Automated pupillometry (Algiscan, ID-MED, Marseille, France) was used to determine pupillary reflex dilation during tetanic stimulation. The maximum intensity of the stimulation value allowed the determination of a pupillary pain index score ranging from 1 (no nociception) to 9 (high nociception): a pupillary pain index (PPI) score of ≤4 was used to reflect adequate pain control. For skin conductance (SC), the number of SC peaks per second (NSCF) was collected concomitantly to tetanic stimulation. An NSCF of ≤0.07 peak/second was used to reflect adequate pain control. Results: Of the 51 included patients, there were 32 with brain injury and 19 receiving deep sedation. Mean PPI score was 5 (Interquartile Range= 2–7); a total of 28 (55%) patients showed inadequate control of the nociceptive stimulation according to the PPI assessment. Only 15 (29%) patients showed a detectable skin conductance, with NSCF values from 0.07 to 0.47/s. No correlation was found between skin conductance algesimeter (SCA)-derived variables and PPI score or pupillary dilation to pain. Conclusions: Detection of inadequate pain control might vary according to the method used to assess nociception in ICU patients. A poor agreement between quantitative pupillometry and skin conductance was observed.

## 1. Introduction

Pain is a frequent event during critical illness; however, its assessment in patients continues to be a challenge for critical care clinicians [1]. In one study, patients on mechanical ventilation who were assessed for pain within two days of intensive care unit (ICU) admission were more likely to receive sedation and dedicated analgesia during painful procedures than patients whose pain was not assessed; this approach resulted in a shorter duration of mechanical ventilation [2]. However, this strategy is challenging in patients with absent movements in reaction to nociceptive stimulation, i.e., those with severe brain injury and/or receiving deep sedation with the use of neuromuscular blocking agents (NMBAs) [3]. Indeed, the two validated clinical instruments to assess pain in non-verbal patients, i.e., the behavioral pain scale (BPS) and the critical care pain observational tool (CPOT), are based on scoring facial expression, movement of the arms, and respiratory patterns during mechanical ventilation (MV) [4]. In ICU patients who are unable to move, the clinical signs of nociception produce atypical and non-quantifiable reactions to noxious procedures, such as face flushing, clenched teeth, clenched fist, and tremor [5,6].

In this context, efforts have been made to develop non-clinical quantification of nociception. Vital signs, such as changes in heart rate, blood pressure, respiratory rate, and oxygen saturation, were found as poor indicators of nociception [1]. Technology using heart rate variability (i.e., the analgesia nociception index), bilateral bispectral index system (BIS), or incorporating various physiologic parameters (i.e., nociception level index) [7,8,9] still requires more validation in the ICU setting. Pupillary reflex dilation using videopupillometer showed promising results regarding nociceptive assessment in the ICU [10,11,12]. The pupillary pain index (PPI), a score derived from pupillary reflex dilatation measurements during a standardized pain stimulation, could anticipate inadequate analgesia during tracheal aspiration in sedated critically ill patients, regardless of the presence of brain injury [13]. The skin conductance algesimeter (SCA) is a device to assess nociception using sympathetic-induced changes in skin conductance [14]; SCA has been explored in the perioperative setting with interesting results [15,16,17]. SCA might be of interest in ICU patients because skin conductance is marginally influenced by circulatory alterations, adrenergic medication, NMBAs, or body temperature [18].

The aim of this study was therefore to compare concomitant measurements of pupillometry and skin conductance during noxious stimulation in critically ill unconscious patients. The primary objective of this pilot study was to compare the number of patients with no evidence of nociception using each technique.

## 2. Materials and Methods

### 2.1. Study Population

In this monocentric, prospective, and observational study conducted between 1 April 2019 and 15 August 2019, adult patients admitted to the ICU of Erasme Hospital, Brussels, Belgium were enrolled if they met the following inclusion criteria: an initial Glasgow coma score (GCS) scale <9 with a motor response <5, the requirement of deep level of sedation (Richmond Agitation-Sedation Scale—RASS of −5), and mechanical ventilation. Patients with known pupillary abnormalities, multiple sclerosis, ocular surgery, severe peri-orbital edema limiting pupillary assessment, pregnancy, or burns were not included. Patients were explored during weekdays, using concomitant measurements of quantitative pupillometry and skin conductance within the first 72 h of their admission to the ICU. Clinical assessment of pain was performed using the BPS; in patients receiving NMBAs, BPS was reported with the score of 3. The level of sedation was measured using the RASS score [19].

### 2.2. Quantitative Pupillometry

Quantitative pupillometry was performed by two operators using an automated pupillometer (NeuroLight Algiscan, ID-MED, Marseille, France). Two pupillary reflexes were explored: the pupil light reflex (PLR) and the pupil dilation reflex during noxious stimulation. To measure PLR, a burst of light of fixed intensity (320 lux) and duration (one second) was emitted by the device to each eye. A minimum duration of one minute was allowed between appraisals of the two pupils to obtain full recovery of baseline pupil diameter after light stimulation. A total of 4 s is needed to evaluate baseline pupil size (mm), pupillary constriction rate (i.e., the difference between baseline and post-stimulation pupil size, expressed as % of constriction from the baseline value), and constriction velocity (CV) (mm/s). Mean values from both eyes were used for baseline assessment. Anisocoria was defined as a difference of at least 1.0 mm between the two eyes [20].

**Figure 1:**
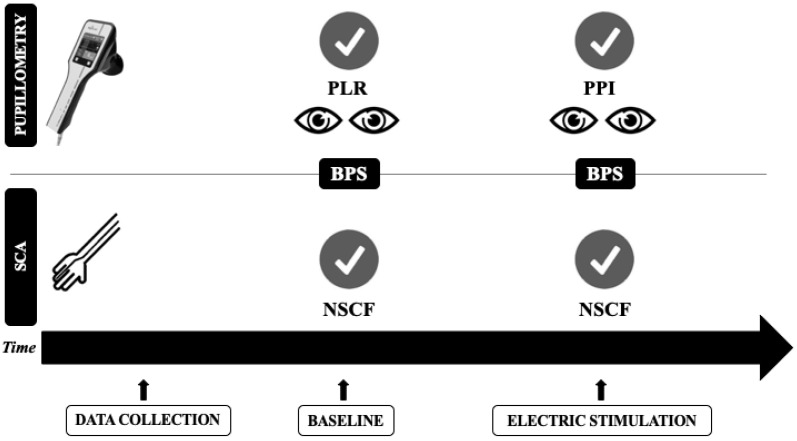
Timeline of the study protocol. PLR = pupillary light reflex; PPI = pupil pain index; NSCF = number of skin conductance fluctuations; BPS = behavioral pain scale; SCA = skin conductance algesimeter.

After baseline assessment (Figure 1), an electrical stimulation with stepwise, gradual intensity (increasing from 10 mA to a maximum of 60 mA) was applied on the left forearm of each patient, using two electrodes linked to the pupillometer. Both pupils were assessed during the stimulation for a total of two pupillometry recordings; the average values of these analyses were calculated. Baseline pupil size (mm), pupillary dilation to pain (%), and the PPI were obtained. This index measures pupil dilation in response to a continuously increasing 1-s electric stimulus from 10 to 60 mA using steps of 10 mA. When an increase of 13% in pupil dilation reflex (PDR) is detected, the stimulus stops, and PPI is calculated by the device; it assigns a score from 1 (pupillary dilation <5% to the maximal stimulation intensity) to 10 (pupillary dilation >13% with 10 mA stimulus) [21]. A PPI score of ≤4 was used to define adequate pain control.

### 2.3. Skin Conductance Algesimeter

Skin conductance was monitored during five minutes at baseline assessment before the tetanic electrical stimulation. A skin conductance monitor (Med-Storm Pain Monitoring System^®^; MED-STORM Innovation AS, Oslo, Norway) was used. Three single use Ag/AgCl electrodes were attached to the palmar surface of the patient’s hand (i.e., the thenar eminence for the current electrode; the hypothenar eminence for the measurement electrode; and just below the second and third digits for the reference electrode), on the opposite site to the pupillometry’s electrodes. The PainMonitorTM, a measurement unit connected with such electrodes, was used to provide a small electrical signal between the electrodes in order to evaluate the skin conductance, which was recorded on a computer with dedicated software to analyze the electrical signal. Skin conductance was measured using an alternating current (66 Hz, 50 mV). The following parameters were collected: (a) the number of skin conductance fluctuations per second (NSCF) or number of peaks/second; (b) average peak (micro-Siemens/second, μSs); (c) average rise (μSs) that is the mean of skin conductance related to the basal sympathetic tone; and (d) area under huge peak (μSs) and area under small peak (μSs). These parameters were automatically calculated by the device and analyzed during a window of 15 s, the update being made second by second. The mean value for each of these variables, representing the 15-s window at baseline and after the stimulation, was extracted for analysis. The cut-off that identifies a normal skin conductance is 0.02 μSs, as showed in previous studies [22,23]; also in addition, a value of NSCF equal or lower than 0.07 peak/second was considered as “absence of pain” while a value of at least 0.27 peak/second was considered as a visual analog scale (EVA) >4, as stated by the manufacturer. Monitoring was not blinded so that the observers could see the skin conductance curve and the values of NSCF and note when artefacts disturbed registration. During the pain stimulation using the automated pupillometry, two markers were inserted on SCA recordings. 

### 2.4. Data Collection

Demographics, medical history, and clinical data on the day of automated pupillometry and SCA assessment were collected. The severity of illness of each patient was assessed using the sequential organ failure assessment (SOFA) score on ICU. Reasons for ICU admission as well as the presence of primary brain injury and concomitant therapies (i.e., vasopressors, renal replacement therapy, or extra-corporeal membrane oxygenation (ECMO)) were also collected. The use of drugs that might interfere with pupillary constriction (i.e., opiates, sedatives, or barbiturates) and of anti-epileptic drugs was also noted. Typical procedures, such as washing, turning the patient, physiotherapy, or invasive procedures were not allowed during the study period. No adjustment of data to reduce the potential sources of bias was performed.

### 2.5. Statistical Analysis

Discrete variables were expressed as counts (percentage) and continuous variables as means ± standard deviation (SD) or median (25th–75th percentiles) as appropriate. The Kolmogorov–Smirnov test were used and histograms and normal-quartile plots examined to verify the normality of distribution of continuous variables. Differences between groups were assessed using a χ-square or Fisher’s exact test for categorical variables, as appropriate, a paired *t*-test or a Wilcoxon rank test for paired continuous variables, and a *t*-test or a Mann–Whitney-U test for continuous independent variables. To test the relationship between automated pupillometry and SCA-derived variables, Pearson’s (or Spearman) correlation analyses were performed, accordingly. The agreement between the two techniques was evaluated by comparing the number of patients with adequate pain control (PPI ≤ 4 and NSCF < 0.07) using Cohen’s Kappa coefficient. An additional analysis comparing patients with and without primary brain injury was also performed. Furthermore, characteristics of patients with concordant and non-concordant results from the two techniques were analyzed. A *p* < 0.05 was considered statistically significant. Statistical analyses were performed using IBM SPSS Statistics 25.0 for Macintosh (Armonk, NY, USA). Using the upper confidence interval for the population variance approach to the sample size calculation, a pilot sample size between 30 and 50 was chosen, corresponding to standardized effect sizes of 0.4 and 0.7 (for 90% power based on a standard sample size calculation) [24].

## 3. Results

### 3.1. Patient Characteristics 

A total of 51 patients were included during the study period; 22 other patients were eligible but not included: 11 admitted during the weekend, 9 refusals to participate, and 2 with previous ocular surgery. Characteristics of the study population are shown in Table 1. Thirty-two patients were admitted with an acute brain injury: post-anoxic coma (*n* = 13), hemorrhagic stroke (*n* = 13), traumatic brain injury (*n* = 5), and status epilepticus (*n* = 1). Other included patients had septic shock (*n* = 4), cardiogenic shock (*n* = 4), hemorrhagic shock (*n* = 2), acute respiratory distress syndrome (*n* = 7), respiratory failure after heart or lung organ transplantation (*n* = 2), and acute chronic liver failure (*n* = 1). The median time from ICU admission to the pain study was 2 (1–4) days. In the study cohort, 39 patients (76%) were receiving opioids (33/39 sufentanil) and 38 (75%) receiving sedatives (propofol, *n* = 29; midazolam, *n* = 4; inhaled sevoflurane, *n* = 2; thiopental, *n* = 1; propofol and sevoflurane, *n* = 1; and propofol and midazolam *n* = 1). Fifteen (29%) patients were treated with a continuous infusion of NMBAs. ICU mortality was 37% (*n* = 19). 

### 3.2. Automated Pupillometry and SCA Assessment

At baseline, mean pupil size was 2.7 (2.2–3.2) mm, PLR was 20 (13–25)% and mean constriction velocity was 1.26 (0.98–2.19) mm/s. Only three patients had anisocoria. In two patients, NSCF was 0.27/s and 0.20/s at baseline, while all others had values of 0 for NSCF and other SCA-derived variables (Table 2). All patients also had a BPS of 3 at baseline and a RASS score of −5.

After the electrical variable stimulation, there were no significant changes in oxygen saturation, while respiratory rate significantly increased from 22 (15–30) to 24 (17–30)/min (*p* < 0.01), mean arterial pressure significantly increased from 83 (71–99) to 86 (71–101) mmHg (*p* = 0.01), and heart rate increased from 81 (71–99) to 83 (72–95) bpm (*p* = 0.04). Among the 36 patients with no NMBA treatment, 7 (19%) showed an increase of BPS from baseline ranged from 4 to 8. Median PPI score after stimulation was 5 (2–7); a total of 28 (55%) patients showed inadequate control of the nociceptive stimulation according to the PPI assessment. Concomitantly, 15 patients had a detectable skin conductance with NSCF ranged from 0.07 to 0.47/s (in Table 3, values pre- and post-stimulation of SC are reported).

**Figure 2:**
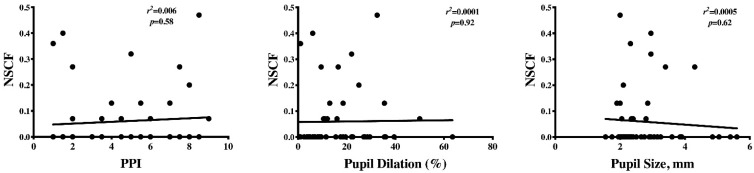
Correlation between the number of conductance fluctuations per second (NSCF) and the pupillary pain index (PPI), the dilation rate (%), and the baseline pupil size (mm) at noxious stimulation.

We found no correlation between PPI score, pupillary dilation to pain and pupil size before stimulation with NSCF (Figure 2), or any of the SCA-derived variables.

A disagreement (i.e., “non-concordant results”) between the two techniques was found in 25/51 patients: 6 had detectable NSCF with PPI ≤ 4 and 19 had high PPI with undetectable skin conductance (Table 4). Thus, the k coefficient of agreement of PPI and NSCF for pain control under a nociceptive stimulation was 0.06. Patients with non-concordant results between the two techniques were older and had a higher body temperature than others (Supplemental Table S1); the proportion of patients with a brain injury was similar between the two groups (15/25, 60% vs. 17/26, 65%, *p* = 0.77).

### 3.3. Subgroup Analysis of Patients with and without Brain Injury

**Figure 3:**
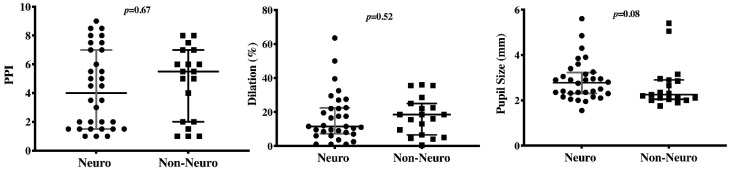
Differences in the pupillary pain index (PPI), the dilation rate (%), and the baseline pupil size (mm) at noxious stimulation between patients with (“neuro”) and without (“non-neuro”) a primary brain injury on ICU admission.

On the day of nociceptive stimulation assessment, patients with brain injury had significantly higher blood pressure and arterial saturation, despite a similar pupil size and a lower constriction rate and constriction velocity at light stimulation compared to others (Table 2). They also received sedative agents less frequently than patients without brain injury. However, no differences at pupillometry or SCA findings were observed after nociceptive stimulation (Figure 3).

## 4. Discussion

In this study including a heterogenous population of critically ill patients on mechanical ventilation, 55% of them had inadequate control of the nociceptive stimulation according to the PPI assessment, and 29% of them had a detectable skin conductance. The two techniques showed poor agreement in the assessment of the response to noxious stimulation.

Critically ill patients are very likely to experience nociception from many causes; however, as many ICU patients are unable to communicate their nociception because of altered consciousness, sedation, or mechanical ventilation, unrelieved pain is a major challenge for clinicians and requires adequate detection. The gold standard in pain evaluation is patient self-reporting, which is not always possible. Current research has shown that the two tools best validated for patients unable to self-report their pain are the BPS and the critical care pain observation tool [25]. However, these scales might present some limitations in those patients with a reduced level of consciousness, in particular when this is secondary to a primary brain injury. Moreover, altered consciousness might per se alter patients’ ability to cooperate during different procedures, which can also exacerbate anxiety and pain. Moreover, the use of sedatives, the presence of severe brain injury, and concomitant NMBAs administration can significantly alter the usefulness of these behavioral scales in ICU patients. As such, reliability and validity of the scales could be challenged in this specific subgroup of patients. In our study, one third of patients received NMBAs, so behavioral pain scales could not be used. Interestingly, changes in systemic physiological variables are associated with pain but the increase rate could be not always easily detectable or not clinically relevant. These observations suggest that it would be almost impossible to assess pain in these complex ICU patients without additional tools that could quantify the response to noxious stimulations.

The use of pupillary reflex dilation has shown some promising findings. During general anesthesia, pupil reflex dilatation has shown greater sensitivity, when compared with other variables such as heart rate and blood pressure, to assess response to noxious stimulation [26,27], and was nicely correlated with self-reported patient’s pain in the post-operative period [28]. In sedated non-brain injured patients, pupillary light reflex distinguished unambiguously between noxious and non-noxious procedures [11] and it was used to predict autonomic response after endotracheal suctioning [13]. In one study, PLR was studied before the start of a noxious stimulation and compared to BPS obtained during stimulation, showing a significant correlation between the two parameters [11]. In a recent study, Vinclair et al. [13] compared the pupillary response obtained during a tetanic stimulation (PPI score) and the BPS response to endotracheal suctioning in sedated non-brain-injured and brain-injured patients; the authors concluded that the nociceptive response to endotracheal suctioning was accurately predicted using the PPI score in these patients, regardless of the presence of brain injury. However, the authors excluded brain-injured patients without preservation of midbrain function as attested by pupillary light reflex measurements (in line with data from post cardiac arrest patients) [29]. In our study, we included different forms of brain-injury, including those suffering from post-anoxic damage and also cortical function at baseline. We found that brain-injured patients, despite a lower constriction rate and constriction velocity at light stimulation than others, showed similar pupillary dilatation to pain when compared to non-brain injury patients, suggesting that PPI assessment could also be effective in this setting. This finding could be explained by the fact that PDR is not mediated directly within the midbrain function (i.e., as the pupillary constriction via the Edinger–Westphal nucleus) but via the cervical sympathetic system, which may remain unaffected by acute brain injury involving the brainstem.

The skin conductance algesimeter is a device that has some potential in ICU patients as its accuracy is minimally biased by confounders, such as shock, medications, or hypothermia, which are frequently observed during critically illness [18]. In one study, Ledowsky et al. found a positive correlation between NSCF during surgery and the numeric rate scale in the post-operative setting [15]. However, the same group of authors found, in another study, that NSCF only partially reflected the changes in plasma noradrenaline levels during surgery and that this parameter was only partially influenced by boluses of fentanyl [16]. In the ICU setting, there are scarce data, in particular on unconscious patients. In one study, SCA indices significantly increased during tracheal suction and patient positioning; also in addition, NSCF negatively correlated with the Ramsay scale [22]. In another study, SCA was more sensitive than cardiovascular, respiratory, or even BIS-derived variables to detect pain [30]. However, deep sedation could affect the intensity of SCA changes during patient’s stimulation. Moreover, in non-sedated patients, NSCF could be influenced by concomitant emotional distress and anxiety rather than by pain alone [23]. Interestingly, all the studies conducted in the ICU excluded patients with neuro-myopathy, patients treated with NMBAs and those with brain injury. As such, we reported for the first time the use of SCA during noxious stimulation in such patients, with a high degree of severity. 

It remains difficult to explain the discrepancy in the number of patients with inadequate analgesia between automated pupillometry and SCA. We can speculate that the two devices have a different sensitivity for pain detection when sedatives and analgesics are used; as this situation is also present in the operative setting, it is possible that the presence of a critical illness, of an inflammatory status, or of brain damage would negatively affect the capacity of skin conductance to adequately assess pain in this setting. Moreover, as critical illness is often associated with microvascular dysfunction, this phenomenon may potentially influence more peripheral release of catecholamines and autonomous nerves than ocular pathways. Moreover, the anatomic pain pathways, which are “tested” by the two monitoring tools, are different and this could also influence our findings. During emotion or pain, skin sympathetic nerves, although under the control of the cerebral cortex, activate palmar and plantar sweat glands that eventually increase skin conductance (i.e., “peak”); this phenomenon is mediated by the acetylcholine via the muscarine receptors [14]. The constrictor pupillae and the dilator pupillae receive opposing parasympathetic and sympathetic innervations; the sympathetic neurons are located in the intermedio-lateral column of the cervico-thoracic spinal cord and project to the noradrenergic postganglionic neurons in the superior cervical ganglion, which innervate the dilator pupillae muscle; the locus coeruleus is also involved in the pupillary control and response to noxious stimulation [31]. These pathways are not involved in the pupil light reflex, which explains why we could not find any association between pupil light reflex at baseline and PPI during pain stimulation. Finally, the SCA showed NSCF data over an average of 15-s time window while tetanic stimulation with pupillometry rarely exceeded 10 s, which could explain the sensitivity in pain detection over a short-time noxious stimulus.

This study had several limitations. First, the number of included patients was relatively small to make appropriate comparison of patients with and without brain injury. Moreover, the presence of patients with post-anoxic brain injury might provide an additional bias on the alterations of pain pathways and additional data on this specific population are required. Second, treatments for sedation and analgesia were not standardized, which might have introduced bias in the interpretation of data. The small number of patients prevented us from making appropriate adjustments. Third, the electrical stimulation was variable in time and/or intensity, i.e., adjusted according to PDR, and patients received therefore different degree of nociception; whether a tetanic stimulation of fixed and constant intensity would have been more appropriate to test nociception between the two monitoring tools remains unknown. Fourth, the effect of opioids on pupillary light reflex might provide an additional bias on the alterations of pain pathways and further studies are necessary to better define this issue. Finally, age is another important factor that can interfere with pupillary constriction or dilation, because the resting size of the pupil progressively decreases after the fourth decade of life; however, we did not adjust our observations on this variable.

## 5. Conclusions

In this study, 55% of patients showed inadequate control of noxious stimulation according to the automated pupillometry assessment while changes in skin conductance were more limited. The two techniques showed a poor agreement in assessing pain in critically ill unconscious patients.

## Figures and Tables

**Table 1:** Characteristics of the study population. Subgroup analysis comparing patients with primary brain injury (“NEURO”) and others is also reported. Data are presented as count (%) or median (Interquartile Ranges—IQRs).

	ALL (*n* = 51)	NEURO (*n* = 32)	NON-NEURO (*n* = 19)
Age, years	60 (49–70)	58 (48–70)	61 (50–70)
Male, *n* (%)	39 (76)	19 (59)	13 (68)
ICU admission to test, days	2 (1–4)	2 (1–4)	2 (1–6)
COMORBID DISEASES			
COPD/asthma, *n* (%)	9 (18)	5(16)	4 (21)
Heart failure, *n* (%)	11 (22)	5 (16)	6 (32)
Hypertension, *n* (%)	27 (53)	15 (47)	12 (63)
Diabetes, *n* (%)	9 (18)	8 (25)	1 (5)
Immunosuppression, *n* (%)	4 (8)	1 (3)	3 (16)
Liver cirrhosis, *n* (%)	3 (6)	1 (3)	2 (10)
Chronic renal failure, *n* (%)	9 (18)	5 (16)	4 (21)
SOFA score on admission	9 (8–12)	9 (8–11)	11 (8–13)
LIFE-SUPPORT THERAPIES			
CRRT, *n* (%)	8 (16)	3 (9)	5 (26)
ECMO, *n* (%)	4 (8)	0 (0)	4 (21) *
DRUGS			
Opioids, *n* (%)	39 (76)	24 (75)	15 (78)
Sedatives, *n* (%)	38 (75)	19 (59)	19 (100) *
NMBAs, *n* (%)	15 (29)	9 (28)	6 (32)
Vasopressors, *n* (%)	34 (67)	18 (56)	16 (84)
Inotropic agents, *n* (%)	5 (10)	1 (3)	4 (21)
OUTCOME			
ICU mortality, *n* (%)	19 (37)	14 (44)	5 (26)

Data are presented as mean ± SD, median (25th–75th percentiles) or count (%). COPD = chronic obstructive pulmonary disease; ICU = intensive care unit; AP = automated pupillometry; SOFA = sequential organ failure assessment; CRRT = continuous renal replacement therapy; ECMO = extracorporeal membrane oxygenator; NMBAs = neuromuscular blocking agents. * *p* < 0.05 “neuro” vs. “non-neuro” patients.

**Table 2:** Characteristics of patients on the day of nociceptive stimulation assessment. Subgroup analysis comparing patients with primary brain injury (“NEURO”) and others is also reported. Data are presented as count (%) or median (IQRs).

	ALL (*n* = 51)	NEURO (*n*= 32)	NON-NEURO (*n* = 19)
VITAL PARAMETERS			
MAP, mmHg	83 (71–99)	92 (72–106)	73 (69–83) *
Heart rate, bpm	81 (71–99)	79 (72–98)	84 (68–101)
Respiratory rate, bpm	22 (15–30)	21 (13–30)	24 (19–30)
Arterial saturation, %	98 (96–100)	99 (96–100)	96 (95–99) *
Temperature, °C	36.8 (36–37.2)	36.9 (35.0–37.3)	36.8 (36.3–37.2)
GCS	3 (3–3)	3 (3–3)	3 (3–3)
BASELINE NOCICEPTION PARAMETERS			
*Pupillometry Values*			
Size, mm	2.7 (2.2–3.2)	2.7 (2.3–3.3)	2.5 (2.1–2.9)
Constriction rate, %	20 (13–25)	19 (11–24)	23 (15–33) *
Constriction velocity, mm/s	1.26 (0.98–2.19)	1.11 (0.71–2)	1.66 (1.20–2.49) *
Anisocoria	3 (6)	3 (9)	0 (0)
*Algesimeter Values*			
Area huge peak,	0 (0–0)	0 (0–0)	0 (0–0)
Peak/sec (NSCF)	0 (0–0)	0 (0–0)	0 (0–0)
Average rise, μSs	0 (0–0)	0 (0–0)	0 (0–0)
Average peak, μSs	0 (0–0)	0 (0–0)	0 (0–0)

Data are presented as mean ± SD, median (25th–75th percentiles) or count (%). MAP = mean arterial pressure; GCS = Glasgow coma scale, NSCF = number of skin conductance fluctuations per second. * *p* < 0.05 “neuro” vs. “non-neuro” patients.

**Table 3:** Algesimeter values pre- and post-stimulation in the 15 patients with detectable NSCF. Data are presented as median (IQRs).

	Pre-Stimulation Values	Post-Stimulation Values
Area huge peak,	0 (0–0)	0.30 (0–1.12) *
Peak/sec (NSCF)	0 (0–0)	0.13 (0.07–0.27) *
Average rise, μSs	0 (0–0)	0.01 (0–0.01) *
Average peak, μSs	0 (0–0)	0.02 (0.01–0.06) *

NSCF = number of skin conductance fluctuations per second. * *p* < 0.05 “neuro” vs. “non-neuro” patients.

**Table 4:** Proportion of patients with adequate or inadequate pain control according to changes in the pupillary pain index (PPI) or to the number of skin conductance fluctuations per second (NSCF). Total of patients = 51.

	PPI ≤ 4 (Adequate Control)	PPI > 4 (Inadequate Control)
NSCF < 0.07 (No Pain Detection)	17	19
NSCF ≥ 0.07 (Pain Detection)	6	9

## Data Availability

The data presented in this study are available on request from the corresponding author.

## Supplementary Materials

The following are available online at https://www.mdpi.com/2076-3425/11/1/109/s1, Table S1: Characteristics of the patients according to concordance in nociception detection between skin conductance algesimeter and automated pupillometry.

## References

[1] Devlin J.W., Skrobik Y., Gélinas C., Needham D.M., Slooter A.J.C., Pandharipande P.P., Watson P.L., Weinhouse G.L., Nunnally M.E., Rochwerg B. (2018). Clinical Practice Guidelines for the Prevention and Management of Pain, Agitation/Sedation, Delirium, Immobility, and Sleep Disruption in Adult Patients in the ICU. Crit. Care Med..

[2] Payen J.F., Bosson J.L., Chanques G., Mantz J., Labarere J., DOLOREA Investigators (2009). Pain assessment is associated with decreased duration of mechanical ventilation in the intensive care unit: A post Hoc analysis of the DOLOREA study. Anesthesiology.

[3] Chanques G., Pohlman A., Kress J.P., Molinari N., De Jong A., Jaber S., Hall J.B. (2014). Psychometric comparison of three behavioural scales for the assessment of pain in critically ill patients unable to self-report. Crit. Care.

[4] Payen J.F., Bru O., Bosson J.L., Lagrasta A., Novel E., Deschaux I., Lavagne P., Jacquot C. (2001). Assessing pain in critically ill sedated patients by using a behavioural pain scale. Crit. Care Med..

[5] Roulin M.-J., Ramelet A.-S. (2014). Behavioral changes in brain-injured critical care adults with different levels of consciousness during nociceptive stimulation: An observational study. Intensive Care Med..

[6] Arbour C., Choinière M., Topolovec-Vranic J., Loiselle C.G., Puntillo K., Gélinas C. (2014). Detecting pain in traumatic brain-injured patients with different levels of consciousness during common procedures in the ICU: Typical or atypical behaviors?. Clin. J. Pain.

[7] Broucqsault-Dédrie C., De Jonckheere J., Jeanne M. (2016). Measurement of heart rate variability to assess pain in sedated criti-cally ill patients: A prospective observational study. PLoS ONE.

[8] Arbour C., Gélinas C., Loiselle C.G., Bourgault P. (2015). An Exploratory Study of the Bilateral Bispectral Index for Pain Detection in Traumatic-Brain-Injured Patients with Altered Level of Consciousness. J. Neurosci. Nurs..

[9] Ben-Israel N., Kliger M., Zuckerman G., Katz Y., Edry R. (2013). Monitoring the nociception level: A multi-parameter approach. J. Clin. Monit. Comput..

[10] Li D., Miaskowski C., Burkhardt D. (2009). Evaluations of physiologic reactivity and reflexive behaviors during noxious pro-cedures in sedated critically ill patients. J. Crit. Care.

[11] Lukaszewicz A.C., Dereu D., Gayat E., Payen D. (2015). The relevance of pupillometry for evaluation of analgesia before noxious proce-dures in the intensive care unit. Anesth. Analg..

[12] Paulus J., Roquilly A., Beloeil H., Théraud J., Asehnoune K., Lejus-Bourdeau C. (2013). Pupillary reflex measurement predicts insufficient analgesia before endotracheal suctioning in critically ill patients. Crit. Care.

[13] Vinclair M., Shilte C., Roudad F., Lavolaine J., Francony G., Bouzat P., Bosson J., Payen J. (2019). Using Pupillary Pain Index to Assess Nociception in Sedated Critically Ill Patients. Anesth. Analg..

[14] Storm H. (2008). Changes in skin conductance as a tool to monitor nociceptive stimulation and pain. Curr. Opin. Anaesthesiol..

[15] Ledowsky T., Bromilow M., Peacdh M.J., Storm H., Hacking R., Schug S.A. (2006). Monitoring of skin conductance to assess postopera-tive pain intensity. Br. J. Anaesth..

[16] Ledowski T., Pascoe E., Ang B., Schmarbeck T., Clarke M., Fuller C., Kapoor V. (2010). Monitoring of intra-operative nociception: Skin conductance and surgical stress index versus stress hormone plasma levels. Anaesthesia.

[17] Storm H., Myre K., Rostrup M., Stokland O., Lien M.D., Raeder J.C. (2002). Skin conductance correlates with perioperative stress. Acta Anaesthesiol. Scand..

[18] Storm H., Fremming A., Odegaard S., Martinsen O.G., Morkrid L. (2000). The development of a software program for analyzing spon-taneous and externally elicited skin conductance changes in infants and adults. Clin. Neurophysiol..

[19] Sessler C.N., Gosnell M.S., Grap M.J., Brophy G.M., O’Neal P.V., Keane K.A., Tesoro E.P., Elswick R.K. (2002). The Richmond Agita-tion-Sedation Scale: Validity and reliability in adult intensive care unit patients. Am. J. Respir. Crit. Care Med..

[20] Couret D., Boumaza D., Grisotto C., Triglia T., Pellegrini L., Ocquidant P., Bruder N.J., Velly L. (2016). Reliability of standard pupillometry practice in neurocritical care: An observational, double-blinded study. Crit. Care.

[21] Ly-Liu D., Reinoso-Barbero F. (2015). Immediate postoperative pain can also be predicted by pupillary pain index in children. Br. J. Anaesth..

[22] Khanna P., Chandralekha C., Pandey R.K., Sharma A. (2018). Pain assessment in the critically ill mechanically ventilated adult pa-tients: Comparison between skin conductance algesimeter index and physiologic indicators. Saudi J. Anaesth..

[23] Günther A.C., Bottai M., Schandl A., Storm H., Rossi P., Sackey P.V. (2013). Palmar skin conductance variability and the relation to stimulation, pain and the motor activity assessment scale in intensive care unit patients. Crit. Care.

[24] Kieser M., Wassmer G. (1996). On the use of the upper confidence limit for the variance from a pilot sample for sample size determi-nation. Biom. J..

[25] Gélinas C., Fillion L., Puntillo K.A., Viens C., Fortier M. (2006). Validation of the critical-care pain observation tool in adult patients. Am. J. Crit. Care.

[26] Larson M.D., Sessler D.I., Washington D.E., Merrifield B.R., Hynson J.A., McGuire J. (1993). Pupillary Response to Noxious Stimulation During Isoflurane and Propofol Anesthesia. Anesth. Analg..

[27] Barvais L., Engelman E., Eba J., Coussaert E., Cantraine F., Kenny G. (2003). Effect site concentrations of remifentanil and pupil response to noxious stimulation. Br. J. Anaesth..

[28] Charier D., Vogler M.C., Zantour D., Pichot V., Martins-Baltar A., Courbon M., Roche F., Vassal F., Molliex S. (2019). Assessing pain in the postoperative period: Analgesia Nociception Index^TM^ versus pupillometry. Br. J. Anaesth..

[29] Ploghaus A., Narain C., Beckmann C.F., Clare S., Bantick S., Wise R., Matthews P.M., Rawlins J.N.P., Tracey I. (2001). Exacerbation of pain by anxiety is associated with activity in a hippocampal net-work. J. Neurosci..

[30] Aslanidis T., Grosomanidis V., Karakoulas K., Chatzisotiriou A. (2018). Electrodermal Activity Monitoring during Endotracheal Suction in Sedated Adult Intensive Care Unit Patients. Folia Medica.

[31] Szabadi E. (2012). Modulation of physiological reflexes by pain: Role of the locus coeruleus. Front. Integr. Neurosci..

