# Supplementary material for: Evaluation of Nociception Using Quantitative Pupillometry and Skin Conductance in Critically Ill Unconscious Patients: A Pilot Study
Source: Brain Sci. 2021 Jan 15;11(1):109. doi: 10.3390/brainsci11010109 (PMC7829933; doi:10.3390/brainsci11010109)
Supplement: Supplementary file 1 [file brainsci-11-00109-s001.pdf]

Supplemental Material

Evaluation of nociception using quantitative pupillometry and skin conductance in critically ill unconscious patients: A pilot study

Sara FRATINO[1 §], Lorenzo PELUSO[1 §], Marta TALAMONTI[1], Marco MENOZZI[1], Lucas Akira COSTA HIRAI[1], Francisco A LOBO[2], Chiara PREZIOSO[1], Jacques CRETEUR[1], Jean-François PAYEN[3#], Fabio Silvio TACCONE[1#]

[§]*equally contributed as first authors*

[#]*equally contributed as senior authors*

[1] *Department of Intensive Care Erasme Hospital*

Université Libre de Bruxelles

Route de Lennik, 808

1070 Brussels (Belgium)

[2] *Department of Anesthesiology*

Centro Hospitalar do Porto, Porto, Portugal.

[3]*University Grenoble Alpes*

Department of Anesthesia and Critical Care

CHU Grenoble Alpes, 38000, Grenoble, France

Supplemental Table 1. Characteristic of the patients according to concordance in nociception detection between Skin Conductance Algesimeter and Automated Pupillometry. Data are presented as mean ± SD, median (25th-75th percentiles) or count (%).

	**CONCORDANT (n=26)**	**NOT CONCORDANT (n=25)**	**p values**
Age (years)	56 (43 - 63)	66 (54 – 71)	0.02
Male, n (%)	15 (58)	17 (68)	0.57
ICU admission to assesmeent, days	2 (1 – 3)	3 (2 – 6)	0.08
**COMORBID DISEASES**			
COPD/Asthma, n (%)	4 (15)	5(20)	0.73
Heart Failure, n (%)	6 (23)	5 (20)	1.00
Hypertension, n (%)	14 (54)	13 (52)	1.00
Diabetes, n (%)	7 (27)	2 (8)	0.14
Immunosuppression, n (%)	3 (12)	1 (4)	0.61
Liver cirrhosis, n (%)	2 (8)	1 (4)	1.00
Chronic renal failure, n (%)	4 (15)	5 (20)	0.73
SOFA score on admission	9 (8-12)	10 (8-12)	0.53
**LIFE-SUPPORT THERAPIES**			

CRRT, n (%)	4 (15)	4 (16)	1.00
ECMO, n (%)	2 (8)	2 (8)	1.00
**DRUGS**			
Opioids, n (%)	20 (77)	19 (76)	1.00
Sedatives, n (%)	21 (81)	17 (68)	0.35
NMBAs, n (%)	10 (39)	5 (20)	0.22
Vasopressors, n (%)	19 (73)	15 (60)	0.38
Inotropic agents, n (%)	3 (12)	2 (8)	1.00
Brain Injury, n (%)	17 (65)	15 (60)	0.77
ICU mortality, n (%)	11 (42)	8 (32)	0.57
**BASAL VITAL PARAMETERS**			
MAP, mmHg	83 (71 – 99)	84 (69 -100)	0.70
Heart Rate, bpm	85 (72 – 107)	79 (70 – 88)	0.13
Respiratory Rate, bpm	22 (13 – 30)	22 (18 – 30)	0.50
Arterial Saturation, %	98 (95 – 100)	97 (96 – 99)	0.69
Temperature, ℃	36.3 (35.0 – 36.9)	37.0 (36.8 – 37.4)	<0.01
GCS	3 (3 - 3)	3 (3 - 3)	0.39
**BASAL NOCICEPTION PARAMETERS**			
Pupillometry Values			

Size, mm	2.7 (2.1 - 3.3)	2.7 (2.4 - 2.9)	0.97
Constriction Rate, %	18 (13 - 24)	23 (15 - 27)	0.21
Constriction Velocity, mm/s	1.22 (1.03 – 1.93)	1.66 (0.98 – 2.36)	0.40
Anisocoria	2 (8)	1 (4)	1.00
Algesimeter Values			
Area huge peak,	0 (0 – 0)	0 (0 – 0)	0.31
Peak/Sec (NSCF)	0 (0 – 0)	0 (0 – 0)	0.96
Average Rise, μSs	0 (0 – 0)	0 (0 – 0)	0.16
Average Peak, μSs	0 (0 – 0)	0 (0 – 0)	0.96

COPD = chronic obstructive pulmonary disease; ICU = intensive care unit; AP = automated pupillometry; SOFA = Sequential Organ Failure Assessment, CRRT = Continuous Renal Replacement Therapy, ECMO = Extracorporeal Membrane Oxygenator, NMBAs = Neuromuscular Blocking Agents, MAP = mean arterial pressure; GCS = Glasgow Coma Scale, NSCF = number of skin conductance fluctuations per second
